# An oxidoreductase from ‘Alphonso’ mango catalyzing biosynthesis of furaneol and reduction of reactive carbonyls

**DOI:** 10.1186/2193-1801-2-494

**Published:** 2013-10-01

**Authors:** Ram Kulkarni, Hemangi Chidley, Ashish Deshpande, Axel Schmidt, Keshav Pujari, Ashok Giri, Jonathan Gershenzon, Vidya Gupta

**Affiliations:** Plant Molecular Biology Unit, Division of Biochemical Sciences, CSIR-National Chemical Laboratory, Pune, 411 008 India; Department of Biochemistry, Max Planck Institute for Chemical Ecology, Beutenberg Campus, Jena, D–07745 Germany; Dr. Balasaheb Savant Konkan Agriculture University, Dapoli, 415712 India; Center of Excellence in Epigenetics, Indian Institute of Science Education and Research (IISER), Pune, 411 008 India

**Keywords:** Detoxification, Enone oxidoreductase, Flavor, *Mangifera indica*, Ripening

## Abstract

Two furanones, furaneol (4-hydroxy-2,5-dimethyl-3(2H)-furanone) and mesifuran (2,5-dimethyl-4-methoxy-3(2H)-furanone), are important constituents of flavor of the Alphonso cultivar of mango (*Mangifera indica*). To get insights into the biosynthesis of these furanones, we isolated an enone oxidoreductase gene from the Alphonso mango. It has high sequence similarity to an alkenal/one oxidoreductase from cucumber (79% identity) and enone oxidoreductases from tomato (73% identity) and strawberry (72% identity). The complete open reading frame was expressed in *E. coli* and the (his)_6_-tagged recombinant protein was purified by affinity chromatography. The purified protein assayed with NADH as a reducing agent converted D-fructose-1,6-diphosphate into furaneol, the immediate precursor of mesifuran. The enzyme was also able to convert two highly reactive carbonyls, 3-buten-2-one and 1-penten-3-one, produced by lipid peroxidation in plants, into their saturated derivatives. Expression profiling in various ripening stages of Alphonso fruits depicted an expression maxima at 10 days after harvest stage, shortly before the appearance of the maximum amount of furanones (completely ripe stage, 15 days after harvest). Although no furanones were detected at the 0 day after harvest stage, significant expression of this gene was detected in the fruits at this stage. Overall, the results suggest that this oxidoreductase plays important roles in Alphonso mango fruits.

## Background

Flavor is one of the most important attributes that decides acceptability of food items that one consumes. The sensation of flavor perceived is usually because of a mixture of many compounds in the food. Nevertheless, some of them dominate the flavor of a particular food item and thus, are themselves capable of eliciting the flavor response in humans similar to that induced by food material. Furanones, which are found in some food products, represent one such dominating class of flavor compounds. In processed food, these compounds are formed as sugar degradation products by Maillard reaction induced by heat treatments (Schwab and Roscher 1997). Furanones are also important as naturally occurring flavor compounds. They are found in many fruits including strawberry, pineapple, raspberry, grapes, tomato, kiwi and mango and many contribute caramel-like flavor notes of these fruits (Schwab and Roscher 1997). In addition to having a sweet and pleasant odour, furanones, especially furaneol and mesifuran, are characterized by a low odour detection threshold (Pino and Mesa 2006). Our previous studies demonstrated that ripe mango fruits contain high amounts of furaneol (4-hydroxy-2,5-dimethyl-3(2H)-furanone) and its methyl ether, mesifuran (2,5-dimethyl-4-methoxy-3(2H)-furanone). The fruits of cultivar Alphonso contained higher amounts of these two compounds than those found in any other cultivar (Pandit et al. 2009a), and ripening of Alphonso fruits was characterized by *de novo* appearance and increase in the level of these furanones (Pandit et al. 2009b). Although furanones are not the most dominant compounds of the Alphonso fruits quantitatively, the low odour detection threshold of furanones makes their contribution to Alphonso mango flavor, about 20-fold higher than that of any other volatile compound, in terms of odour units (Kulkarni et al. 2012).

Considering such crucial involvement of furanones in determining the flavor of mango and other fruits, the enzymes involved in the biosynthetic pathway of furanones can have huge commercial potential for production of flavor chemicals. Nonetheless, biosynthesis of furaneol and mesifuran has until now been studied only in strawberry and tomato. Earlier studies on strawberry showed that out of several radiolabeled substrates fed to the ripening strawberry fruits, D-fructose-1,6-diphosphate had the highest rate of incorporation into furaneol (Roscher et al. 1998). This, along with the other studies (Schwab 1998; Wein et al. 2001) confirmed D-fructose-1,6-diphosphate as a natural precursor of furanones in the plants. Further studies carried out to understand the biosynthesis of furaneol in plants indicated that D-fructose-1,6-diphosphate is first converted by an unknown enzyme into an unstable intermediate, 4-hydroxy-5-methyl-2-methylene-3(2H)-furanone (HMMF). The furaneol forming enzyme, enone oxidoreductase, highly similar to the NAD(P)H:quinone oxidoreductase, then reduces the exocyclic α, β unsaturated bond of HMMF, resulting in the formation of furaneol (Raab et al. 2006). Enone oxidoreductases from strawberry and tomato were capable of converting various derivatives of HMMF, substituted at the methylene group, into their respective saturated products (Raab et al. 2006; Klein et al. 2007). The presence of HMMF has also been detected in the fruits such as pineapple and raspberry suggesting that the biosynthetic pathway of furanones might be similar in various plants. Recently, an oxidoreductase, highly similar to strawberry and tomato enone oxidoreductase, capable of reducing α, β-unsaturated carbonyls, has been characterized from cucumber (Yamauchi et al. 2011). Apart from the pathway contributed by the plant, biosynthesis of furaneol in strawberry has also been suggested to occur in collaboration with enzymes from an epiphytic bacterium, *Methylobacterium extorquens* (Zabetakis 1997; Koutsompogeras et al. 2007; Verginer et al. 2010; Zabetakis et al. 2006).

Furaneol further contributes to the fruit flavor by converting into its methyl ether, mesifuran. The enzyme responsible for the formation of mesifuran is known only from strawberry (Wein et al. 2002) and it was shown to be an S-adenosyl methionine-dependent methyl transferase that methylates the hydroxyl group of furaneol. To get insights into the biosynthesis of furaneol and mesifuran in mango, another fruit containing high amounts of these furanones, we here report the isolation and characterization of a cDNA that encodes an enone oxidoreductase activity, catalyzing the biosynthesis of furaneol in ripe fruits of the Alphonso mango.

## Results

### Isolation of complete open reading frame of the enone oxidoreductase

In order to isolate genes involved in the biosynthesis of furaneol, we sought sequences that might encode the enone oxidoreductase activity. Degenerate primers designed based on the alignment of the database entries designated as quinone oxidoreductases from *Fragaria x ananassa* (AY048861), *Vigna radiata* (U20808) and *Helianthus annuus* (AF384244) were used for isolating similar sequence from the ripe fruits of Alphonso mango. Sequences of the fragments obtained showed high similarity to those of quinone oxidoreductases from the other plants. For obtaining the full-length open reading frame of this gene, gene specific primers were designed and were used for rapid amplification of cDNA ends (RACE). The 5′ and 3′ RACE fragments thus obtained again showed high similarity to the respective terminal regions of the mRNA sequences of other quinone oxidoreductases. Based on these alignments, terminal primers were designed and the full-length cDNA of a putative mango enone oxidoreductase (*MiEO*-*Mangifera indica* enone oxidoreductase) was obtained by amplification of the cDNA prepared from the ripe fruits.

**Figure 1:**
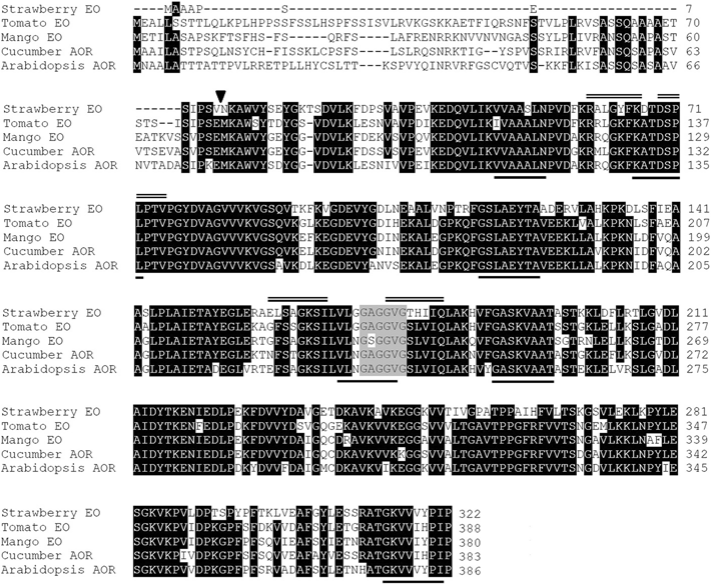
**Alignment of the** ***in silico*** **translated sequence of the** ***Mi*** **EO with the closest characterized sequences from other plants.** Regions of the alignment corresponding to the nucleotide sequence used for designing degenerate primers are marked by the lines below the alignment, and that used for designing gene specific primers for RACE are indicated by double lines above the alignment. The conserved NAD(P)H-binding domain is highlighted in the gray colour. The arrow head indicates truncation site for removing the putative chloroplast targeting sequence.

The complete open reading frame (ORF) of the *MiEO* was 1143 bp long and was flanked by 40 bp and 115 bp UTR at the 5′ and 3′ ends, respectively. The ORF encoded a protein having 381 amino acids, calculated molecular weight of 40.6 kD and pI of 8.61. The *in silico* translated amino acid sequence of the *Mi*EO was most similar to the chloroplastic alkenal/one oxidoreductase (AOR) from *Cucumis sativus* (*Cs*AOR) (79% identity) (Yamauchi et al. 2011), enone oxidoreductase (EO) from *Solanum lycopersicon* (*Sl*EO) (73% identity) (Klein et al. 2007), EO from *Fragaria x ananassa* (*Fa*EO) (72% identity) (Raab et al. 2006) and AOR from *Arabidopsis thaliana* (*At*AOR) (71% identity) (Yamauchi et al. 2011). The putative amino acid sequence of the *Mi*EO showed presence of the conserved GxGxxG domain which is involved in binding with NADP (Edwards et al. 1996) (Figure 1).

### *Mi*EO catalyzes synthesis of furaneol and reduction of unsaturated ketones

**Figure 2:**
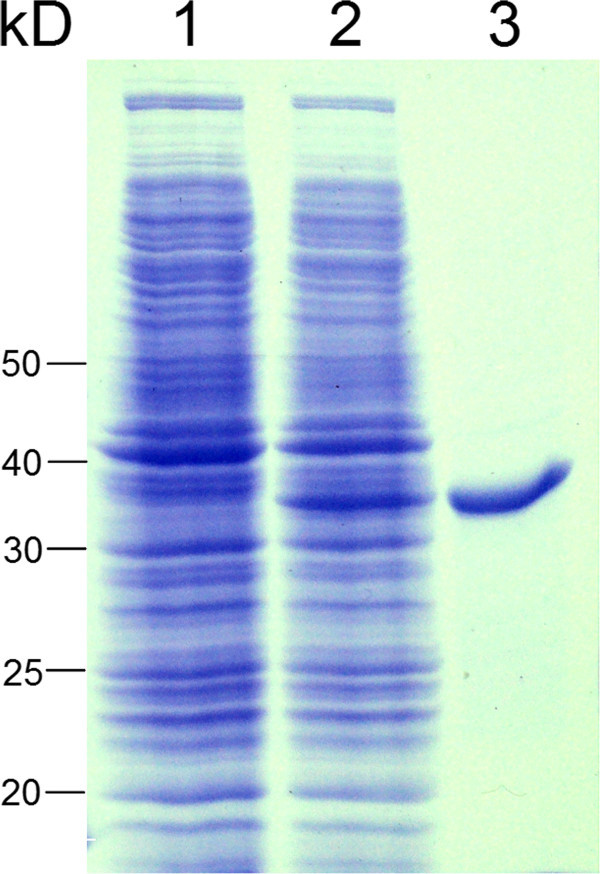
**SDS-PAGE profile of the crude lysate of the cells carrying plasmid with reverse-oriented insert (lane 1), crude lysate of the cells carrying** ***Mi*** **EO construct (lane 2) and the purified recombinant** ***Mi*** **EO protein (lane 3).** Sizes of the proteins (kDa) in the molecular marker are indicated on the left side.

**Figure 3:**
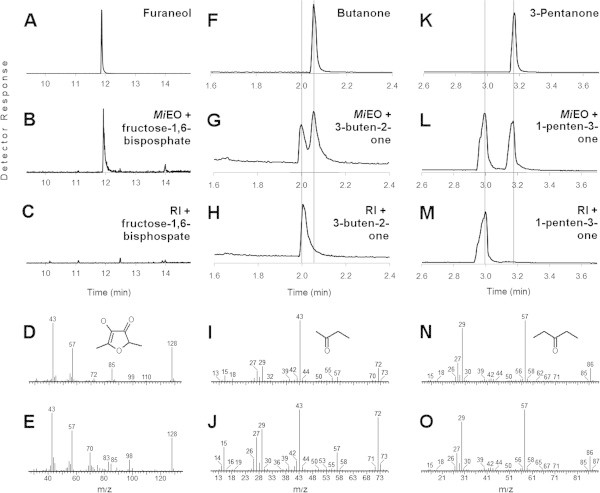
**GC-MS analysis of the enzymatic assays of the** ***Mi*** **EO with various substrates, viz, D-fructose-1,6-diphosphate (A-E), 3-buten-2-one (F-J) and 1-penten-3-one (K-O).** The chromatograms represented are of the authentic standards of the expected products **(A**, **F** and **K)** and the assays of the protein expressed from the *Mi*EO **(B**, **G** and **L)** and the plasmid carrying reverse oriented insert **(C**, **H** and **M)** with the respective substrates (RI: reverse insert). For the assays with D-fructose-1,6-diphosphate, the chromatograms **(A**, **B** and **C)** represent trace of *m/z* 128 corresponding to the molecular ion of furaneol; whereas, for the assays with the other substrates, total ion chromatograms were monitored. The spectra represented are of the authentic standards of the expected products, *viz*, furaneol **(D)**, butanone **(I)** and 3-pentanone **(N)** and of the peaks obtained in the *Mi*EO assays at the time corresponding to the expected products **(E**, **J**, **O)**. The presence of *m/z* 70, 83 and 98 in **(E)** was because of the contaminating co-eluent which was also detected at the same time in **(C)**.

Similar to the *Cs*AOR, the *At*AOR and the *Sl*EO, N-terminal region of the *in silico* translated *Mi*EO was characterized by the presence of the putative chloroplast targeting peptide as revealed by analysis of the sequence by ChloroP program, suggesting that the *Mi*EO protein might be localized in the chloroplast. To get functional insights into the *Mi*EO, the complete open reading frame without the 5′-nucleotide stretch corresponding to the putative chloroplast-targeting peptide (Figure 1) was cloned into the pCRT7/NT-TOPO vector and the ORF was expressed in *E. coli* as 6xhis-tagged protein (Figure 2). The enzymatic activity of the purified recombinant *Mi*EO protein was assessed using D-fructose-1,6-diphosphate as a substrate and NADH as a reducing agent and the product formation was examined by gas chromatography–mass spectrometry. As has been reported previously (Raab et al. 2006), in the initial experiments, furaneol was detected in assays with the protein expressed from the plasmid having the reverse-oriented insert, indicating that this activity was due to the background proteins from the *E. coli* expression system. However, increasing the stringency of the wash solution to 40 mM imidazole during purification of the protein by affinity chromatography resulted in diminished oxidoreductase activity originating from *E. coli*. The *Mi*EO protein purified and assayed with D-fructose-1,6-diphosphate clearly showed the presence of furaneol as a reaction product in the GC-MS analysis (Figure 3). Although D-fructose-1,6-diphosphate is not a direct natural precursor of furaneol, an enzyme from strawberry, *Fa*EO, was also shown to be able to covert D-fructose-1,6-diphosphate to furaneol (Raab et al. 2006). Detection of the furaneol in the assays of purified *Mi*EO with D-fructose-1,6-diphosphate as substrate combined with the absence of furaneol in the assays of boiled protein confirmed the furaneol forming activity of the *Mi*EO. In view of the fact that the *Mi*EO also showed high sequence similarity to the *Cs*AOR, which catalyzes reduction of unsaturated carbonyls; this activity of the *Mi*EO was also assayed with substrates such as 3-buten-2-one and 1-penten-3-one, which are highly reactive fatty acid derived chemicals found in the plants including mango (Pino et al. 2005; Pino and Mesa 2006; Tamura et al. 2001). Surprisingly, the *Mi*EO was also able to reduce these unsaturated ketones into their respective saturated derivatives, *viz*., 2-butanone and 3-pentanone, with NADH as a reducing agent (Figure 3).

### *MiEO* is expressed throughout ripening of the mango fruits

**Figure 4:**
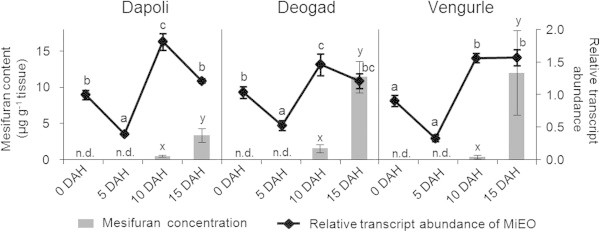
**Mesifuran content and relative transcript abundance of the** ***Mi*** **EO in the ripening fruits of the Alphonso mango from the three cultivation localities, Dapoli, Deogad and Vengurle in India (DAH: days after harvest).** Letters indicate the significance of ANOVA (p ≤ 0.01) for comparison between the ripening stages for the levels of mesifuran **(x**, **y**, etc.**)** and the relative transcript abundance of the *Mi*EO **(a**, **b**, etc.**)**; the values having different letters are significantly different from each other.

To get insights into the possible physiological role of the *Mi*EO in fruits of the Alphonso mango, transcripts of the *MiEO* were measured through various ripening stages. The highest expression of the *MiEO* was detected at the 10 DAH (days after harvest) stage of the ripening fruits (Figure 4). There was a reduction in the expression of the *MiEO* during transition from 10 DAH to 15 DAH (ripe) fruits in contrast to the increase in the concentration of furanones (furaneol and mesifuran) observed along this transition. Although furanones are completely absent in the unripe fruits (0 DAH), the expression level of the *MiEO* was only about 1.5-fold lower than that in the ripe fruits and there was about two-fold reduction in expression during the transition of fruit from 0 to 5 DAH stage.

Our previous study has demonstrated the existence of geographic variation in the flavor volatiles of the Alphonso mangoes (Kulkarni et al. 2012). Out of the three localities which were studied for the content of volatiles, Dapoli was characterized by the lowest amount of mesifuran in the ripe fruits; whereas, for the 10 DAH stage, the highest amount of mesifuran was detected in the fruits from Deogad (Kulkarni et al. 2012). To know if there is any contribution of the *Mi*EO to such geographic variation, expression of the *MiEO* was also analyzed in the ripening fruits of mangoes from these cultivation locations. Although there were some differences between the localities for the level of the *MiEO* transcripts, the pattern could not be correlated with the varied mesifuran content among the localities.

## Discussion

This study was initiated with the intention of studying biosynthesis of furanones and its regulation in Alphonso mango. Mango is the only third plant after strawberry and tomato in which the biosynthesis of furaneol has been studied. In all the three species, a gene encoding enone oxidoreductase, the last enzyme in biosynthesis of furaneol has been characterized. Since ripe fruits of Alphonso mango contain high amounts of furaneol and mesifuran (Pandit et al. 2009b), and *Mi*EO has been shown to be producing furaneol by *in vitro* assays in our study, the most likely *in planta* function of *Mi*EO could be the biosynthesis of furaneol. In the ripening fruits of Alphonso mango, the peak level of furanones is detected at the ripe stage (15 DAH) (Pandit et al. 2009b; Kulkarni et al. 2012); whereas, the highest expression of *MiEO* was seen at 10 DAH stage among the stages of fruit ripening analyzed in the present study. This discrepancy can be attributed to the fact that peak transcript level usually precedes the highest accumulation of the corresponding metabolite. However, in strawberry it was shown that the expression of a similar gene, the *FaEO*, was highly correlated with the furanone accumulation during the fruit development (Raab et al. 2006). It is important to note, nevertheless, that strawberry is a non-climacteric fruit whereas mango is a climacteric fruit and there are considerable differences in the ripening physiology and the expression patterns of various genes in these two kinds of fruits (Lee et al. 2010). Another difference observed was that unlike strawberry, the precursor of furaneol, HMMF, was not detected in the mango fruits (Klein et al. 2007). It could be because of 5 fold lower concentration of furanones observed in mango as compared to strawberry (Kulkarni et al. 2012; Pandit et al. 2009b; Wein et al. 2002) and short half-life of HMMF. These observations also suggest that in addition to the biosynthesis of furaneol, *Mi*EO might also be involved in other biochemical reactions.

The *Mi*EO showed high sequence identity with some other enzymes from the other plants. One such enzyme, the *Cs*AOR from *Cucumis sativus*, has been reported to be involved in scavenging the highly reactive unsaturated carbonyl chemicals (Yamauchi et al. 2011). Interestingly, *Mi*EO was also able to catalyze reduction of unsaturated carbonyls *in vitro*. Both of the substrates used in this study, 3-buten-2-one and 1-penten-3-one, and their respective saturated derivatives, butanone and 3-pentanone have been reported in mango fruits (Pino et al. 2005; Pino and Mesa 2006; Tamura et al. 2001). Thus, *Mi*EO might function in planta to scavenge these unsaturated carbonyls.

In conclusion, the *Mi*EO identified and characterized in the present study might have oxidoreductase function leading to furanone biosynthesis as well as scavenging reactive carbonyl molecules during ripening of mango fruits. Detailed characterization of the *Mi*EO, with respect to transgenic expression in model plants would shed more light on the possibility of the multifunctional nature of this gene. The potential application of this gene for biotechnological production of flavor as well as for producing the plants having enhanced resistance towards the oxidative stress also needs to be explored.

## Materials and methods

### Plant material

Mature fruits of mango (cv. Alphonso) were harvested from four plants each from three localities, Dapoli (orchard of Konkan Agriculture University) (N17°45′ E73°11′), Deogad (orchard of Konkan Agriculture University) (N16°31′ E73°20′) and Vengurle (private orchard) (N15°51′ E73°39′) in India. Fruits were put in the hay after harvesting, carried to the laboratory and allowed to ripe at ambient temperature. Fruits were cut at the intervals of five days after harvest, immediately frozen in liquid nitrogen and stored at -80°C until further use, giving rise to four ripening stages, 0, 5, 10 and 15 days after harvest (DAH) from each of the three localities.

### RNA isolation and cDNA synthesis

RNA was isolated as described earlier (Pandit et al. 2007). Reverse transcription was carried out over 1 μg of DNase treated RNA using Enhanced Avian RT First Strand Synthesis Kit (Sigma, St. Louis, MO, USA).

### Isolation of enone oxidoreductase cDNA

Based on the conserved regions in the nucleotide sequences of enone oxidoreductase genes from other plants reported in the NCBI database, following degenerate primers were designed (forward1: 5′-GTKGTKGCTGCWKCYVTTAAYC-3′, forward2: 5′-AARGMYAYYGAYTCTCCYYTRC-3′, forward3: 5′-GGVWSWTTRGCWGARTAYACHGC-3′, forward4: 5′-GTTYTRRRWGGHGCTGGKGGWGTTGG-3′, reverse1: 5′-GRATSGGRTAYAYRACYACYTTYCC-3′, reverse2: 5′-GCYYTHTCHSKYTSYCCWAYTGC-3′, reverse3: 5′-RGTRGCTGCTAYYTTDGAWGCACC-3′) and used for amplification of the cDNA prepared from the ripe fruits of the Alphonso mango in all the possible combinations. The gene specific primers (forward1: 5′-CGAAGACAGGGCAAGTTCAAGGC-3′, forward2: 5′-GATTCTCCCCTCCCGACTGTTCC-3′, reverse1: 5′-GGTGTTGGAAGCTTGGTGATTCAG-3′, reverse2: 5′-GGGTTCTCTGCTGGTAAATCTATTCT-3′) designed based on the sequence of the amplified fragments, were used for the rapid amplification of cDNA ends (RACE) using the SMART™ RACE cDNA Amplification Kit (Clontech, USA). Based on the alignments of the RACE fragments with the nucleotide sequences of enone oxidoreductase from the other plants, primers corresponding to the terminal regions of the mRNA were designed (forward: 5′-ATGAAAGCGTGGGTGTATGGAG, reverse: 5′-TTAAGGAATTGGGTATATAACCACC-3′), and used for PCR with the mango cDNA as a template. The PCR products were eluted from the agarose gel, ligated in pGEM-T Easy vector (Promega, Madison, WI, USA) and the ligation reactions were transformed in *E. coli* cells (Top10). Positive colonies were identified by colony PCR and presence of the desired insert was confirmed by sequencing.

### Expression cloning and recombinant expression in *E. coli*

Full-length sequence of *Mangifera indica* enone oxidoreductase (*MiEO*) was amplified from the cDNA prepared from ripe mango fruits using Advantage 2 PCR Enzyme System (Clontech, USA) and the terminal primers as described above. The resulting fragments were cloned in the pCRT7-NT/TOPO expression vector (Invitrogen, USA), ligation reaction was transformed in the *E. coli* cells (Top10, Invitrogen, USA) and the transformants were selected on the LB-agar medium containing 100 μg ml^-1^ ampicillin. Correct orientation of the insert was confirmed by carrying out a PCR using forward T7 promoter primer and reverse gene specific primer, as well as by sequencing. The recombinant plasmid thus obtained was transformed in BL21(DE3) cells (Invitrogen, USA), for recombinant expression. Starter culture (5 ml) grown for 48 hr at 18°C was used as inoculum for the expression in 100 ml LB media. After induction with 1 mM IPTG at 0.6 OD_600_, cultures were grown for 24 hr at 18°C. The cell pellet obtained after centrifugation was suspended in the buffer containing 25 mM MOPSO (pH 7.2) and 10% (v/v) glycerol. The cells were lysed by sonication and the 6xhis-tagged recombinant proteins were purified by passing the cleared lysate through Ni-NTA spin columns (Qiagen, Germany) following the manufacturer’s instructions. Elution was carried out with the buffer containing 250 mM imidazole, 25 mM MOPSO (pH 7.2) and 10% (v/v) glycerol. Both, the crude lysate and the purified protein were checked for the presence and size determination of the recombinant protein by SDS-PAGE.

### Assay for enzymatic activity

Purified *Mi*EO protein was incubated overnight at 30°C with 60 mg D-fructose-1,6-diphosphate and 3 mg NADH in 1 ml buffer containing 25 mM MOPSO and 10% glycerol (pH 7). The products formed were purified by solid phase extraction using the DSC-18 column having capacity of 3 ml (Sigma, USA). The SPE column was sequentially equilibrated with acetonitrile and assay buffer. After passing the incubation mixture from the column, the products were eluted with dichloromethane and were analyzed by GC-MS. For the assays with 3-buten-2-one and 1-penten-3-one, 0.5 and 1.0 μl of these chemicals, respectively, instead of D-fructose-1,6-diphosphate were individually added to 200 μl of similar assay reaction and the reactions were incubated at 30°C for 75 min. The products were extracted using 100 μm polydimethylsiloxane SPME fiber which was inserted in the vial after 45 min of equilibration and the extraction was carried out for 30 min. The product separation was carried out on the GsBP-5MS column (General Separation Technologies, USA) having the dimensions of 30 m × 0.32 mm i.d. × 0.25 μm film thickness. Oven temperatures for the assay with D-fructose-1,6-diphosphate were programmed from 40°C for 5 min, raised to 220°C at 10°C min^-1^ and held isothermal for 5 min; whereas, those for the assays with 3-buten-2-one and 1-penten-3-one were programmed from 35°C for 9 min, raised to 100°C at 5°C min^-1^ and further to 300°C at 20°C min^-1^ and held isothermal for 2 min. Injector and detector temperatures were 150 and 250°C, respectively. Helium was used as a carrier gas at flow rate 1 ml min^-1^. Mass spectra were obtained using Clarus 500 (Perkin Elmer) gas chromatograph–mass spectrometer at 70 eV with a scan time of 0.2 s. For the assays with D-fructose-1,6-diphosphate, only the ion of m/z 128 of furaneol was monitored to enhance the selectivity of the detection. In a separate analysis total ion chromatograph was also recorded and was used for examining spectra of the furaneol formed in the test assays. For the other two substrates, total ion chromatograms were monitored throughout the study.

### Quantitative real time PCR

Quantitative real time PCR was performed with Brilliant SYBR Green QPCR Master mix (Stratagene). Elongation factor 1α (EF1α) was used as an internal control (Pandit et al. 2010) with the primers described earlier (Pandit et al. 2010). The primers used for the *MiEO* were, forward: 5′-AGGTGCTGTAACACCTCCAGGCT -3′ and reverse: 5′-CCTGGCTGAAAGGAAATGGCCCC -3′.At least three amplicons were cloned and sequenced to verify the primer specificity. Transcript abundance was quantified with a Mx3000P Real Time PCR Thermocycler (Stratagene, USA) using a program with 45 cycles of 95°C for 30 s, 63°C for 30 s and 72°C for 30 s, followed by a melting curve analysis of transcripts. The relative transcript abundance for the raw stage (0 DAH) was considered 1 and the fold change for rest of the tissues was calculated. Each value was represented by four biological and at least two technical replicates.

## Abbreviations

DAH: Days after harvest
HMMF: 4-hydroxy-5-methyl-2-methylene-3(2H)-furanone
ORF: Open reading frame
RACE: Rapid amplification of cDNA ends
SPME: Solid phase microextraction.
